# Multiparametric bone MRI targeting aides lesion selection for CT-guided sclerotic bone biopsies in metastatic castrate resistant prostate cancer

**DOI:** 10.1186/s40644-023-00644-w

**Published:** 2023-12-15

**Authors:** Ricardo Donners, Ines Figueiredo, Daniel Westaby, Dow-Mu Koh, Nina Tunariu, Suzanne Carreira, Johann S. de Bono, Nicos Fotiadis

**Affiliations:** 1 Department of Radiology, Royal Marsden Hospital, Downs Road, Sutton, SM2 5PT UK; 2 Department of Radiology, University Hospital Basel, Petersgraben 4, Basel, 4031 Switzerland; 3 The Institute of Cancer Research, 15 Cotswold Road, Sutton, SM2 5NG UK; 4 The Royal Marsden NHS Foundation Trust, Downs Road, Sutton, SM2 5PT UK; 5 Department of Interventional Radiology, Royal Marsden Hospital, 203 Fulham Rd, London, SW3 6JJ UK

**Keywords:** Neoplasms, Image-guided biopsy, Computer tomography, Genomics, Bone marrow

## Abstract

**Background:**

Bone biopsies in metastatic castrate-resistant prostate cancer (mCRPC) patients can be challenging. This study’s objective was to prospectively validate a multiparametric bone MRI (mpBMRI) algorithm to facilitate target lesion selection in mCRPC patients with sclerotic bone disease for subsequent CT-guided bone biopsies.

**Methods:**

20 CT-guided bone biopsies were prospectively performed between 02/2021 and 11/2021 in 17 mCRPC patients with only sclerotic bone disease. Biopsy targets were selected based on MRI, including diffusion-weighted (DWI) and T1-weighted VIBE Dixon MR images, allowing for calculation of the apparent diffusion coefficient (ADC) and the relative fat-fraction (rFF), respectively. Bone marrow with high DWI signal, ADC < 1100 µm^2^/s and rFF < 20% was the preferred biopsy target. Tumor content and NGS-feasibility was assessed by a pathologist. Prognostic routine laboratory blood parameters, target lesion size, biopsy tract length, visual CT density, means of HU, ADC and rFF were compared between successful and unsuccessful biopsies (p < 0.05 = significant).

**Results:**

Overall, 17/20 (85%) biopsies were tumor-positive and next-generation genomic sequencing (NGS) was feasible in 13/18 (72%) evaluated samples. Neither laboratory parameters, diameter, tract length nor visual CT density grading showed significant differences between a positive versus negative or NGS feasible versus non-feasible biopsy results (each p > 0.137). Lesion mean HU was 387 ± 187 HU in NGS feasible and 493 ± 218 HU in non-feasible biopsies (p = 0.521). For targets fulfilling all MRI selection algorithm criteria, 13/14 (93%) biopsies were tumor-positive and 10/12 (83%) provided NGS adequate tissue.

**Conclusions:**

Multiparametric bone MRI can facilitate target lesion selection for subsequent CT-guided bone biopsy in mCPRC patients with sclerotic metastases.

**Trial registration:**

Committee for Clinical Research of the Royal Marsden Hospital registration number SE1220.

**Supplementary Information:**

The online version contains supplementary material available at 10.1186/s40644-023-00644-w.

## Introduction

A significant proportion of metastatic castrate-resistant prostate cancer (mCRPC) patients harbor clinically actionable genome aberrations, which allow for targeted therapies [1]. Tumor biopsies are essential for treatment decision-making based on individual tumor genomics. However, obtaining high quality tissue samples, containing sufficient tumor cells for advanced molecular analyses, such as next-generation genomic sequencing (NGS), can be challenging in mCRPC patients.

In mCRPC, bone is by far the most common metastatic site and up to 50% of patients show only skeletal disease [2–4]. This is problematic, as bone metastases are often viewed as suboptimal biopsy targets compared with soft-tissues [5]. Even when performed under CT-guidance, there is a significant chance that a bone biopsy will be tumor-negative or contain only few tumor cells, omitting NGS [5–9].

There are no established guidelines for optimal biopsy target selection in mCRPC patients with bone metastases. Generally, lytic (low CT-attenuation) lesions are preferred over sclerotic (high CT-attenuation) lesions [6, 7, 9–11]. However, the former are less common in mCRPC [4]. Reported biopsy success rates for malignant tumor diagnosis and genomic sequencing from sclerotic prostate cancer bone metastases are relatively poor, ranging between 33% and 54% [5, 8, 9]. This warrants further research for improvement of these results.

Imaging plays a key role for biopsy target lesion selection, but CT lacks specificity for discrimination between active and treated sclerotic bone disease. MRI on the other hand, is the gold standard for assessment of bone marrow conditions. In a retrospective study, a simple multiparametric bone MRI (mpBMRI) algorithm using diffusion-weighted MRI (DWI) signal intensity (SI), apparent diffusion coefficient (ADC) and relative fat-fraction (rFF) percentages reliably identified active bone metastases yielding a tumor-positive and NGS feasible biopsy result [12].

The purpose of this study was to test and validate the mpBMRI target lesion selection algorithm in a prospective setting to facilitate target lesion selection for subsequent CT-guided bone biopsies in mCRPC patients with sclerotic disease.

## Materials and methods

This prospective study was approved by the institutional review board and performed as part of multiple phase I and II clinical trials, which required tumor biopsies and were run at our institution. Biopsies were obtained as per respective trial protocol and no additional interventions were performed nor extra samples obtained for this bone biopsy study. Each patient provided written informed consent for trial inclusion, the CT-guided bone biopsy and research related use of medical data.

### Patients

Patients were consecutively recruited between 15/02/2021 and 10/11/2021. Study inclusion criteria were: mCRPC with a histopathological diagnosis, patients were fit for CT-guided bone biopsy procedure and MRI, presence of accessible, sclerotic bone lesions, and availability of written informed consent. Exclusion criteria were: lack of fitness for CT-guided bone biopsy, contraindications to percutaneous biopsy according to contemporary guidelines with particular attention to coagulation status [13, 14], general MRI contraindications and lack of sclerotic bone lesions. When patients had accessible soft-tissue or lytic bone metastases, these were chosen as biopsy targets regardless of the presence of sclerotic bone disease and patients were excluded from this study.

Patient blood parameters obtained within four weeks prior to biopsy, including alkaline phosphatase (ALP) in Units/L, lactate dehydrogenase (LDH) in Units/L, hemoglobin (Hb) in g/L, platelets x10^9^/L, International Normalized ratio (INR) and prostate specific antigen (PSA) levels in µg/L as well as Gleason scores were recorded.

### Imaging

**Fig. 1:**
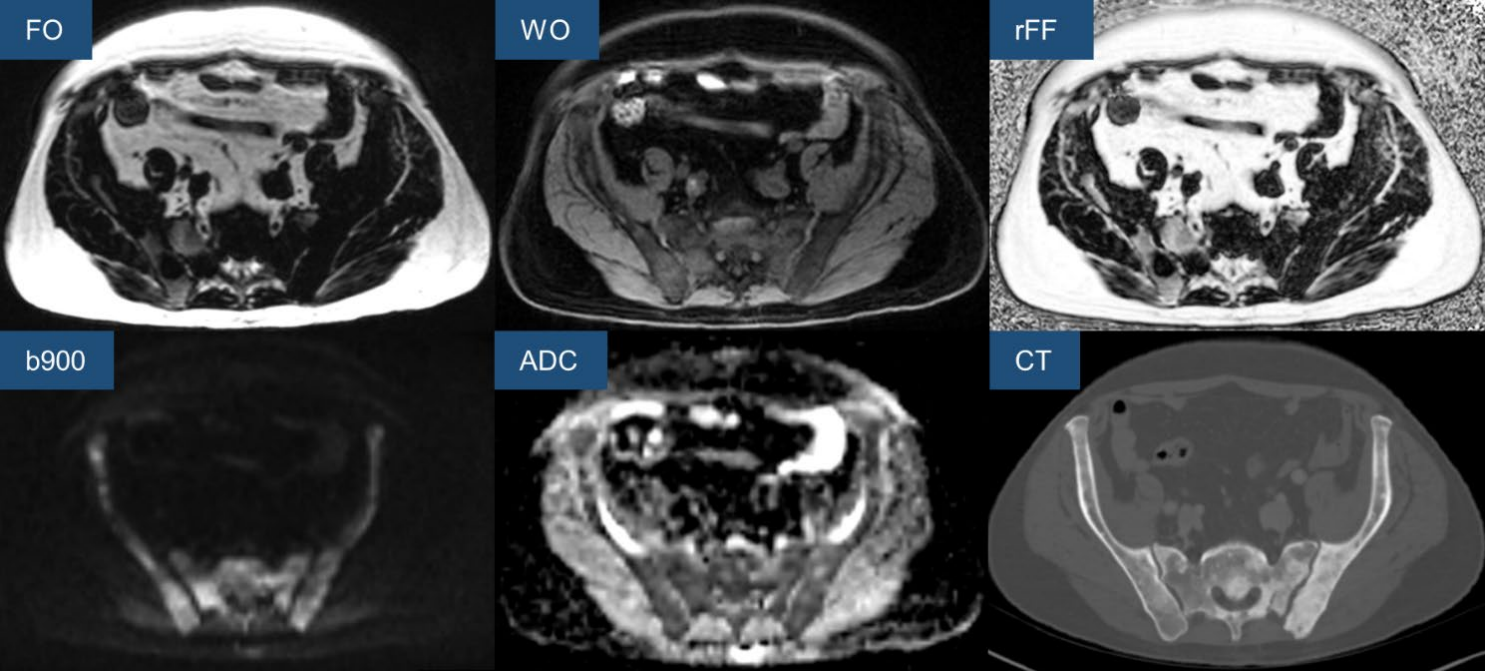
Multiparametric bone MRI imaging protocol for CT-guided bone biopsy target lesion selection including DWI and T1-weighted, gradient-echo Dixon images, CT of the same area for comparison, FO – fat-only Dixon image, WO – water-only Dixon image, rFF – relative fat fraction, b900 – high b-value DWI with corresponding ADC map

Whole-body (WB) mpBMRI, including free-breathing DWI and breath-hold T1-weighted 2-point volume interpolated breath-hold examination (VIBE) Dixon sequences, was acquired as part of the regular disease staging protocol for each patient. When WB-mpBMRI within 3 months prior to the planned biopsy procedure was not available, dedicated limited field-of-view mpBMRI of the likely biopsy target site (such as the pelvis) determined by available imaging, including prior WB-mpBMRI studies, was performed. Sequence parameters for the institute’s 1.5T MRI scanner (MAGNETOM Sola, Siemens Healthineers) are summarized in Table 1. From the VIBE Dixon fat-only (FO) and water-only (WO) images, relative fat fraction (rFF) images were calculated as rFF = FO/(FO + WO) (Fig. 1).

**Table 1:** Diffusion-weighted and Dixon MRI parameters

Parameter	DWI	T1-weighted VIBE Dixon
Plane	axial	axial
Slice thickness (mm)	5	5
b-values	50, 600, 900	-
Field of view (mm)	430 × 390	430 × 390
Acquisition matrix	134 × 108	256 × 180
Repetition time (ms)	6240	7.14
Echo time (ms)	73	2.39
Number of averages	3–3 – 6 (b50, b600, b900)	1
Flip angle	90°	20°
Bandwidth (Hz/pixel)	1964	470
Acquisition time (min:s)	3:51	0:19

VIBE – volume-interpolated breath-hold examination

### Imaging evaluation and target lesion selection

A diagnostic and interventional radiology fellow with three years of experience in mpBMRI of malignant bone disease, competent in performing CT-guided bone biopsies, evaluated all imaging prior to the biopsy. MpBMRI was scrutinized for bone metastases in accessible location, which would be recommended as biopsy targets according to the bone biopsy lesion selection algorithm. Bone biopsies in lesions with high DWI signal, mean ADC < 1100 µm^2^/s and mean rFF < 20% were selected to offer high diagnostic yield and sufficient material for NGS [12]. Consequently, a target lesion deemed likely to provide high tumor-tissue yield needed to fulfill all of the following MRI criteria:

1. lack of suppression i.e. high signal on DWI.
2. mean ADC < 1100 µm^2^/s.
3. mean rFF < 20%.

**Fig. 2:**
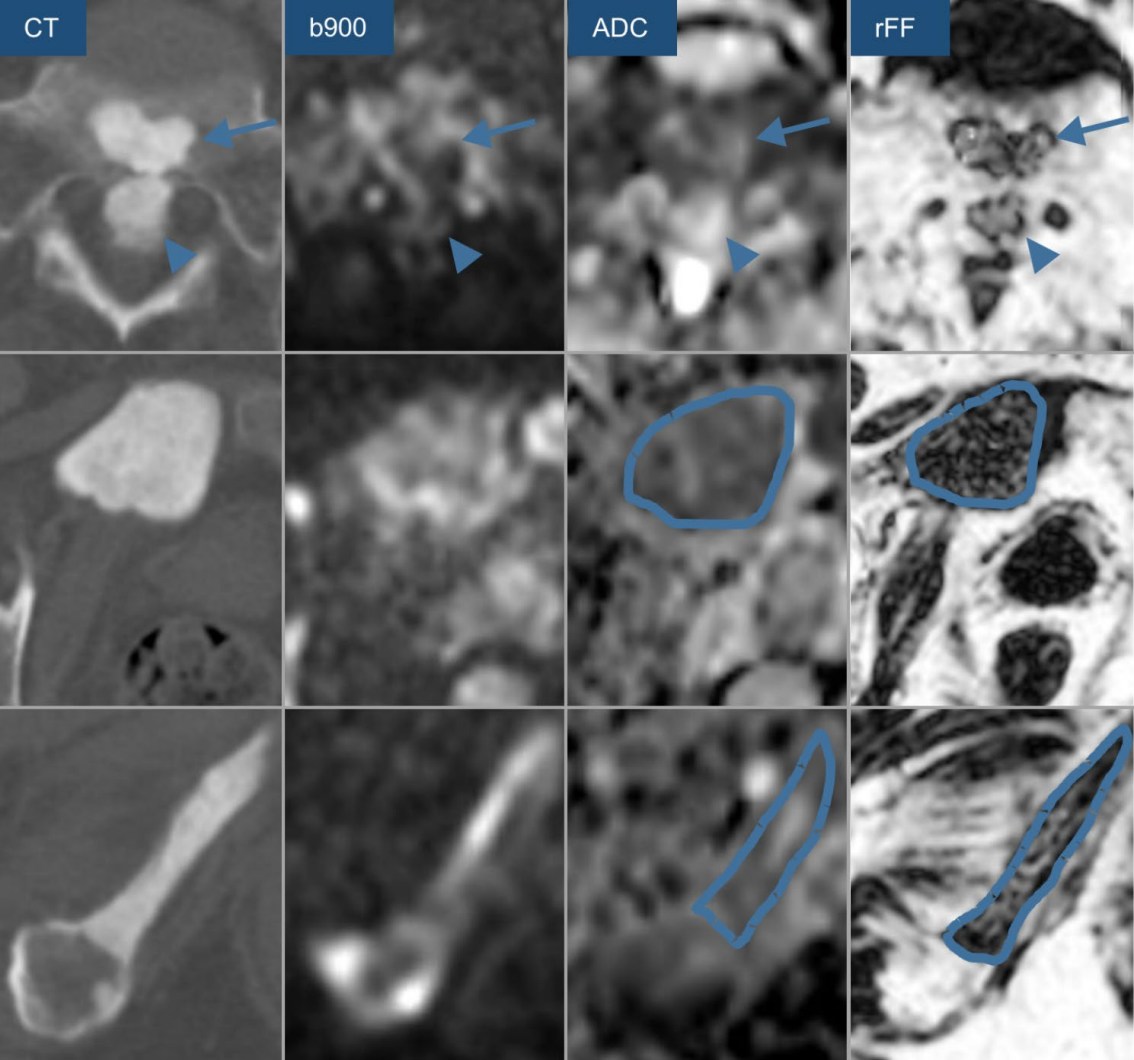
CT-guided bone biopsy target lesion selection process based on multiparametric bone MRI, considering high b-value DWI (b900) signal intensity (SI) ADC and rFF in a metastatic castrate resistant 74-year-old prostate cancer patient with multifocal sclerotic bone metastases, CT images for comparison. The anterior metastasis in the first row (arrow) shows low DWI SI, the posterior lesion (arrowhead) displays high ADC (> 1100 µm^2^/s) and high rFF (> 20%) and was thus not fit for biopsy. The pubic bone metastasis in the second row shows high DWI SI, ADC < 1100 µm^2^/s and rFF < 20% and was biopsied, biopsy provided sufficient malignant tumor cells for next-generation genomic sequencing. The ischial metastases in the third row displays high DWI signal, an ADC < 1100 µm^2^/s, but an rFF of 31% and was thus not considered for biopsy

The target selection process for a patient with multiple sclerotic bone lesions is visualized in Fig. 2. When no accessible metastasis fulfilling all three mpBMRI selection criteria was available, a high DWI signal bone lesion with ADC and rFF values close to the desired thresholds was selected for biopsy. Targets were chosen in consensus with a senior consultant radiologist, with 10-years of experience in mpBMRI of mCRPC. Target lesions were highlighted on the department’s picture archiving and communication systems (PACS, IDS7 Version 22.1, Sectra, Sweden). To minimize procedural risks, pelvic ring lesions were preferred when possible.

### Biopsy procedure

A specialized interventional radiologist, who had been performing CT-guided bone biopsies for 15 years or a supervised interventional radiology fellow performed the biopsies included in this study. MpBMRI with the previously determined target lesion suggestion was reviewed immediately before the procedure.

Patients provided written informed consent for the CT-guided bone biopsy and possible complications and concerns were discussed. The patient was placed in the CT gantry (Somatom Definition Edge, Siemens Healthineers). Positioning was adapted according to the target site, optimizing patient comfort where possible.

The WHO checklist was completed and the procedure site was marked on the patient skin, placing an x-ray grid for orientation. A 3 mm thin slice CT scout encompassing the grid was performed and the needle access position was planned. Patients received 100% oxygen via nasal cannula and intravenous conscious sedation using midazolam and fentanyl under constant monitoring of vital parameters.

**Fig. 3:**
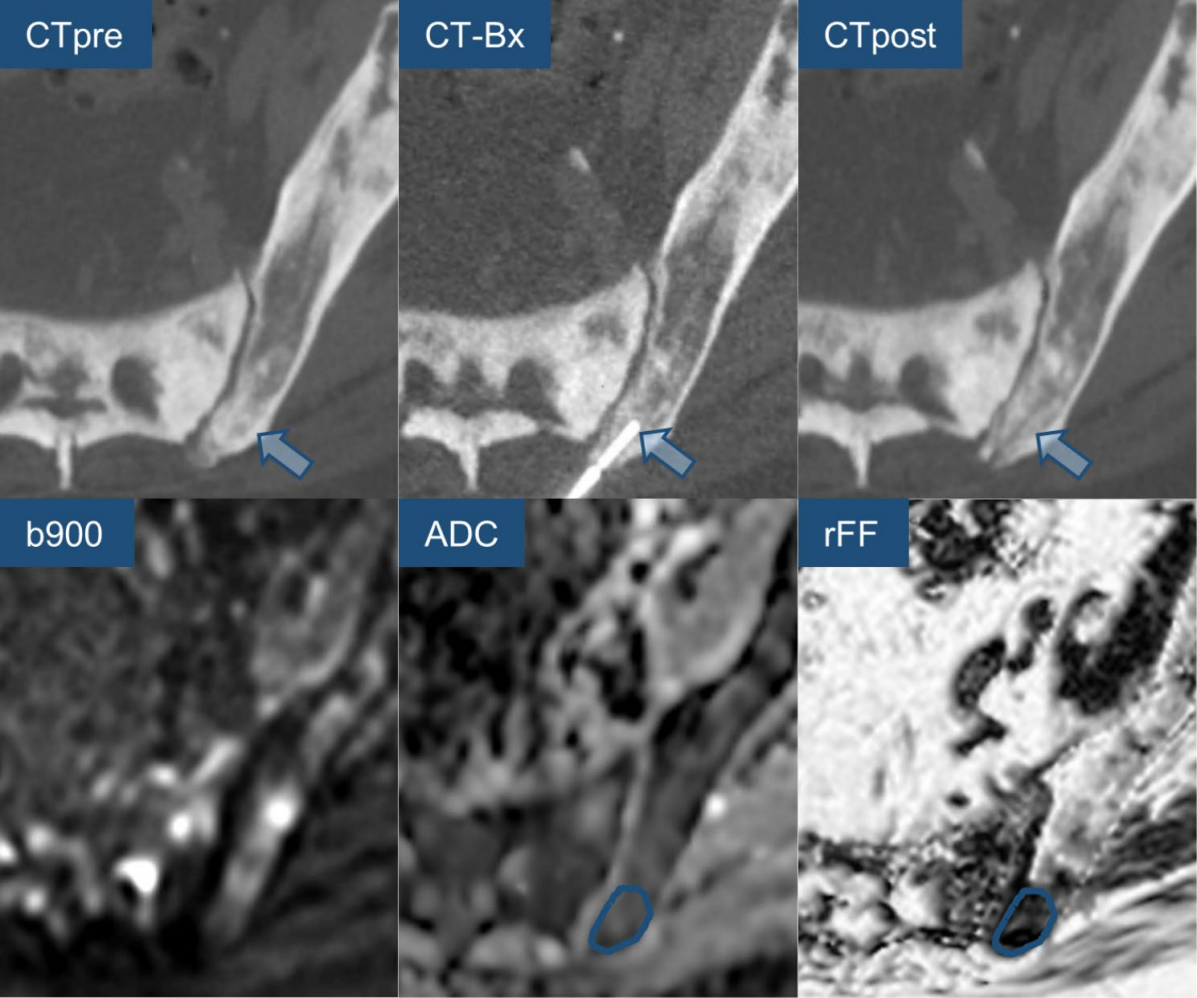
CT-guided bone biopsy in a 86-year old prostate cancer patient, CT before biopsy (CTpre) displays diffuse sclerosis, the chosen target area according to the b900 DWI, ADC and rFF is indicated by the arrow, the biopsy-CT (CT-Bx) and post-interventional control spiral (CTpost) show biopsy needle and biopsy tract in the target area, note how the majority of the sclerotic pelvic bone displays high signal on the rFF image

Lidocaine 1% was applied to the skin and periosteum with CT imaging confirming the correct needle path as planned to the biopsy target. Madison™ (Merit Medical, USA) 11G and 13G biopsy needle systems were used. Bone biopsies were performed under CT-guidance. Biopsy samples were handed to the pathologist and fixed. The biopsy needle systems were removed, dressing was applied to the access site, and a spiral CT scan was performed to rule out immediate complications (Fig. 3**)**. Patients remained under surveillance with bed rest for three hours in the recovery area following the procedure and were then discharged safely home.

Needle gauge (G), length of the biopsy tract and number of bone biopsy samples obtained were recorded. The former was determined by the interventionalist prior to the procedure, with a general preference for a larger diameter needle to provide larger samples. For smaller bone calibre and areas with greater potential risk, such as the sternum, a smaller calibre needle was chosen. The number of biopsy samples was determined by the present member of the pathology team, instructing on the specific number of samples required per patient as per trial protocol as well as by the interventionalist, who performed a visual inspection of the samples. After the biopsy cores were obtained, samples were fixed for 24–30 h and decalcified in an EDTA solution for two days. Bone biopsy samples were deemed suitable for NGS when showing at least 150 cancer cells on H&E stained, 2 μm thick specimen slices. 10 × 6 μm sections were prepared for sequencing. Biopsy specimens containing less than 150 tumor cells on H&E stained slices were deemed not suitable for NGS by the pathologist. When NGS was performed, presence of mutations and copy number variations were recorded.

### Statistics

Statistical analyses were performed using commercially available software (IBM SPSS Statistics Version 25, IBM Corp. Armonk, New York, USA). Qualitative, nominal scaled variables were compared using chi-squared tests. Quantitative, continuous variables were compared using Mann-Whitney-U tests. A p-value < 0.05 was deemed statistically significant.

## Results

Seventeen mCRPC patients with exclusively sclerotic bone metastases underwent 20 CT-guided bone biopsies and were prospectively included in this study. No procedure-related complications were encountered. Median age of the study population was 69 years (range 53–83 years) at the time of biopsy.

Median time interval between mpBMRI and CT-guided bone biopsy was 26 days. 17/20 (85%) biopsies were tumor-positive. Two samples were not evaluated for NGS feasibility as NGS results were already available from earlier biopsies. In total, NGS was feasible in 13/18 (72%) tissue samples. Five biopsies provided insufficient tumor-cell content. NGS results were available for 12 biopsies, as the remaining sample was deemed feasible, but NGS was not performed.

NGS identified tumor genome mutations in 10/12 (83%) samples. Functional importance was rated as pathogenic in 8 samples according to the ClinVar or American College of Medical Genetics and Genomics reporting recommendations. Copy number genome variations were identified in 7 (58%) cases **(**Table 2**)**.

**Table 2:** Biopsy next-generation genomic sequencing results

StudyID	Somatic Mutations			Copy Number Variations
	*Gene*	*Mutation*	*Functional Impact*	
2	SRC TP53	p.P171L p.R158H	Uncertain Uncertain	AR amplification
5	EGFR NOTCH2 TP53	p.A289V p.C342R p.M237I	Uncertain Likely pathogenic Pathogenic	MAP2K1, PTEN deep deletion AR, FANCB amplification
6	CDK12	p.G162W	Uncertain	MAP2K1, PTEN deep deletion AR, FANCB amplification
9	TP53	c.258 + 1G > A	Pathogenic	-
10	FANCG WT1	C308-1G p.H359D	Pathogenic Uncertain	-
12	-			PIK3R1, PTEN deep deletion
14	-			MAP2K4, NOTCH1, PTEN deep deletion AR amplification
15	PIK3CA FANCA	p.E545K p.S132R	Pathogenic Uncertain	-
16	PDGFRA TP53	p.I317T p.R273C	Uncertain Pathogenic	-
17	XPC	c.104-2 A > G	Pathogenic	-
19	AR ATM BLM FANCC FANCF MAP3K1 MSH6 RECQL4	p.T878A p.T1743I p.F663I p.R555Q p.A186V p.D1170del p.R1334W p.G120R	Pathogenic Likely pathogenic Uncertain Likely benign Benign Uncertain Pathogenic Likely benign	AR, ATRX, RECQL4 amplification
20	BRCA2 PIK3CA TP53	p.I1859Kfs*3 p.E545K p.R282W	Pathogenic Pathogenic Pathogenic	AR amplification

Functional impact of each somatic mutation is rated as per ClinVar and American College of Medical Genetics and Genomics reporting recommendations, AR – androgen receptor, PTEN – phosphatase and tensin homologue

11G bone biopsy needles were used in 18/20 biopsies and 13G needles for the remaining two. Between two and six samples were obtained for each biopsy, the median number was three biopsy cores.

### Laboratory parameters

Median values of laboratory parameters are summarized in Table 3. There was no significant difference between laboratory parameter measurements in tumor-positive compared with negative biopsies (each p > 0.266) nor between NGS-feasible and non-feasible biopsies (each p > 0.153).

**Table 3:** Median routine blood parameters and tumor marker

	Tumor			NGS		
Parameter	*Positive*	*Negative*	*P*	*Feasible*	*Not-feasible*	*P*
AlkPh U/L	195	274	0.266	267	191	0.588
LDH U/L	241	182	0.634	241	182	0.153
HB g/L	110	119	0.874	119	110	0.323
Platelets x 10^**9**^**/L**	268	255	0.791	268	321	0.301
PSA µg/L	244	198	0.705	244	129	0.391
Gleason score	7.5	7	0.925	8	7.5	1.000

NGS – Next-generation genomic sequencing, AlkPH - alkaline phosphatase, LDH - lactate dehydrogenase, HB – hemoglobin, PSA – prostate specific antigene, P < 0.05 = significant

### Lesion diameter and biopsy tract length

There were no significant differences in lesion diameter between tumor-positive (mean 22 mm) and negative biopsies (mean 47 mm, p = 0.152) nor between NGS-feasible (mean 23 mm) and non-feasible biopsies (39 mm, p = 0.401). Analogously, no significant differences were found regarding biopsy tract length in tumor-positive (mean 17 mm) and negative (mean 25 mm, p = 0.222) nor between NGS-feasible (mean 17 mm) and non-feasible (mean 25 mm, p = 0.322) biopsies.

### CT lesion appearance and attenuation

Twelve biopsied lesions were classified as predominantly mildly sclerotic and eight as predominantly densely sclerotic. Overall, 11/12 (92%) mildly sclerotic biopsies and 6/8 (75%) biopsies of predominantly densely sclerotic lesions were tumor-positive (p = 0.306). NGS was feasible in 10/12 (83%) mildly sclerotic lesions and 3/5 (60%) densely sclerotic lesions (p = 0.137).

Mean HU of lesions with tumor-positive biopsies was 398 ± 190 HU and 483 ± 181 HU in tumor-negative biopsies (p = 0.711). Regarding NGS feasibility, mean HU was 387 ± 187 HU in NGS-feasible and 493 ± 218 HU in lesions with NGS non-feasible biopsies (p = 0.521).

### Multiparametric bone MRI target lesion selection

Lesions were only chosen for biopsy, when failing to suppress and consequently showing high signal on DWI. Quantitative mpBMRI mean ADC and mean rFF percentage measurements for all included biopsies are shown in Table 4.

**Table 4:** Multiparametric bone MRI ADC and relative fat fraction (rFF) percentage values of the study cohort

Study ID	Site	Mean ADC in µm^2^/s	Mean rFF in %	mpbRMI target recommendation	Tumor	NGS
1	Os ilium	567	25	No	negative	not feasible
2	Os ilium	855	18	Yes	positive	feasible
3	Os pubis	846	24	No	positive	not feasible
4	Sternum	664	13	Yes	positive	n.a
5	Os ilium	706	8	Yes	positive	feasible
6	Os ilium	736	11	Yes	positive	feasible
7	Sternum	720	12	Yes	positive	n.a.
8	Lumbar vertebra	610	21	No	negative	not feasible
9	Sternum	724	18	Yes	positive	feasible
10	Os ilium	567	23	No	positive	feasible
11	Os ilium	701	11	Yes	negative	not feasible
12	Lumbar vertebra	679	22	No	positive	feasible
13	Os ilium	877	11	Yes	positive	feasible
14	Os ilium	882	8	Yes	positive	feasible
15	Os ilium	567	23	No	positive	feasible
16	Os ilium	762	11	Yes	positive	feasible
17	Os ilium	764	11	Yes	positive	feasible
18	Lumbar vertebra	542	19	Yes	positive	not feasible
19	Os ilium	955	18	Yes	positive	feasible
20	Os ilium	910	19	Yes	positive	feasible

rFF – relative fat fraction, mpBMRI – multiparametric bone MRI, NGS – next-generation genomic sequencing

Overall, 14/20 biopsies fulfilled all of the three criteria of the target lesion selection algorithm (high DWI SI, ADC < 1100 µm^2^/s, rFF < 20%). Among these lesions, 13/14 (93%) biopsies were tumor-positive and 10/12 (83%) provided sufficient material for NGS. This includes eleven mildly and three densely sclerotic lesions. Six bone marrow lesions lacked the “adequate target” mpBMRI selection criteria, and displayed mean rFF slightly larger than 20% (range 21 – 25%). Among these, 4/6 (60%) biopsies were tumor-positive and 3/6 (50%) biopsies provided sufficient malignant tumor cells for NGS.

## Discussion

In this prospective proof-of-principle pilot study we have shown that utilizing mpBMRI for sclerotic bone target selection in mCRPC patients for subsequent CT-guided biopsy results in high biopsy success rates. 85% of biopsies were tumor-positive and the NGS feasibility rate was 72%. Among lesions which fulfilled all three mpBMRI target selection criteria, namely high DWI signal, ADC < 1100 μm/s^2^ and rFF < 20%, 93% of biopsies were tumor-positive and the NGS feasibility rate was 83%.

In previous studies, a 53% tumour-positive and 45% sequencing success rate were described for CT-guided sclerotic bone biopsies [8, 9]. By comparison, the tumour-positivity (85%) and NGS feasibility rate (72%) were substantially improved in our prospective pilot cohort.

Holmes et al. described significantly improved bone biopsy success in predominantly radiolucent compared with sclerotic lesions, with 18/19 (95%) lytic lesion biopsies being tumor-positive [8]. By comparison, only 20/38 (53%) sclerotic bone biopsies were tumor-positive, including 5/15 (33%) predominantly densely sclerotic and 15/23 (65%) predominantly subtle sclerotic lesions. Other researchers found 50% of all bone biopsy cores in 23 prostate cancer patients to be tumour positive and RNA isolation feasible in 14/31 (45%) of prostate cancer sclerotic bone biopsies [5, 9]. Radiolucent, lytic bone metastases are generally chosen over sclerotic bone metastases for CT-guided biopsies [6, 7, 15]. However, lytic bone metastases are often absent in mCRPC patients [4]. For this reason and to provide clinical evidence for mCRPC patients with exclusively sclerotic disease, we excluded lytic metastases from the presented study. Using a previously established mpBMRI targeting algorithm, utilizing DWI SI, ADC and rFF, we achieved very good diagnostic and NGS success rates, while only including sclerotic bone lesions. Our approach led to some improvement over the previously published CT-guided bone biopsy diagnostic (85% versus 76%) and NGS (72% versus 71%) success rates in cancer patients at our institution [12]. Several factors may account for lack of a more substantial increase in biopsy success when comparing this prospective study with the previously published retrospective analysis in our department: First and foremost, the previous analysis was performed independently of CT appearance. It was not limited to sclerotic bone lesions, but included lytic disease as well. Additionally, the 76% tumor-positive and 71% NGS feasibility rate in this previous study were already towards the upper end of published biopsy success rates and limit room for improvement.

More recently, studies suggested that PSMA targeting may improve tumor-tissue yield from CT-guided bone biopsies in prostate cancer patients, but CT attenuation of biopsied lesions was not reported in the available data and needs to be evaluated in the future [16, 17].

Overall, our study results validate the use of mpBMRI for target lesion selection in mCRPC patients with sclerotic bone metastases. Further research to permit translation of our findings to other centers, especially with regard to the reproducibility of quantitative mpBMRI target selection thresholds is needed. Eventually, our suggested approach could lead to a paradigm shift for mCRPC patients with only sclerotic bone metastases: Sclerotic bone disease is commonly regarded as an unsuitable biopsy target for obtaining tumor samples adequate for NGS. The increased tumor tissue yield when using mpBMRI for biopsy target selection may allow these patients to benefit from personalised oncology treatments, including biomarker-driven and molecularly targeted therapies. NGS revealed somatic genome mutations or copy number alterations in all analyzed samples, highlighting the potential patient benefit.

We recognize the small study population as a major limitation for this prospective study. However, previously published prospective CT-guided bone biopsy studies aiming to optimize target lesion selection feature similar size study cohorts. Published analyses of sclerotic bone lesion biopsies in less than 30 patients are frequently used for reference and decision-support [7, 9]. The comparison with these previous studies suggests significant benefit of chosing bone biopsy targets using mpBMRI. As mpBMRI is now part of our preinterventional work-up, no comparison cohort was available for the presented study. There may be a role for MRI-guided procedures, allowing for improved visualisation of marrow lesions. Lesion selection parameters, especially the ADC, depend on the imaging parameters including the selection of b-values. While higher b-values may improve lesion conspicuity they lead to decreased SNR and image quality. The protocol presented in this study adheres to the contemporary recommendation for WB-MRI of prostate cancer bone disease, facilitating translation to other centres [18]. We believe our results supply principal evidence in favor of using mpBMRI to optimize CT-guided bone biopsy target lesion selection applicable to a wider population of prostate cancer patients. This was a single center study. Larger multi-center studies, optimizing parameters across sites and hardware vendors are needed to achieve sufficient technical validation.

## Conclusion

In conclusion, mpBMRI can facilitate target selection in mCPRC patients with sclerotic bone metastases for subsequent CT-guided biopsy. This approach resulted in high diagnostic biopsy yield and feasibility of NGS in this patient group. We recommend mpBMRI as part of the pre-interventional imaging work-up.

### Electronic supplementary material

Below is the link to the electronic supplementary material.

**Supplementary Material 1:** CT-guided, sclerotic bone biopsies in prostate cancer patients, contemporary literature results for CT-guided sclerotic bone biopsies

## Data Availability

Study data are available upon reasonable request.

## Abbreviations

FO: fat–only
NGS: next–generation genomic sequencing
mCRPC: metastatic castrate–resistant prostate cancer
MpBMRI: Multiparametric bone MRI
rFF: relative fat–fraction
VIBE: volume interpolated breath–hold examination Dixon
WB: Whole–body
WO: water–only
