# Supplementary material for: Multiparametric bone MRI targeting aides lesion selection for CT-guided sclerotic bone biopsies in metastatic castrate resistant prostate cancer
Source: Cancer Imaging. 2023 Dec 15;23:121. doi: 10.1186/s40644-023-00644-w (PMC10724964; doi:10.1186/s40644-023-00644-w)
Supplement: Supplementary file 1 — Supplementary Material 1: CT-guided, sclerotic bone biopsies in prostate cancer patients, contemporary literature results for CT-guided sclerotic bone biopsies [file 40644_2023_644_MOESM1_ESM.docx]

**Multiparametric bone MRI targeting aides lesion selection for CT-guided sclerotic bone biopsies in metastatic castrate resistant prostate cancer**

Author Affiliations

Ricardo Donners, MD, ricardo.donners@usb.ch, Department of Radiology, Royal Marsden Hospital, Downs Road, Sutton SM2 5PT, UK, Department of Radiology, University Hospital Basel, Petersgraben 4, 4031 Basel, Switzerland

Ines Figueiredo, The Institute of Cancer Research, 15 Cotswold Road, Sutton SM2 5NG, UK.

Daniel Westaby, MD, The Institute of Cancer Research, 15 Cotswold Road, Sutton SM2 5NG, UK

Professor Dow-Mu Koh, MD, Cancer Research UK Cancer Imaging Centre, The Institute of Cancer Research, 15 Cotswold Road, Sutton SM2 5NG, UK; Department of Radiology, Royal Marsden Hospital, Downs Road, Sutton SM2 5PT, UK.

Nina Tunariu, MD, Cancer Research UK Cancer Imaging Centre, The Institute of Cancer Research, 15 Cotswold Road, Sutton SM2 5NG, UK; Department of Radiology, Royal Marsden Hospital, Downs Road, Sutton SM2 5PT, UK.

Suzanne Carreira, PhD, The Institute of Cancer Research, 15 Cotswold Road, Sutton SM2 5NG, UK, The Royal Marsden NHS Foundation Trust, Downs Road, Sutton SM2 5PT, UK.

Professor Johann S. de Bono, PhD, The Institute of Cancer Research, 15 Cotswold Road, Sutton SM2 5NG, UK, The Royal Marsden NHS Foundation Trust, Downs Road, Sutton SM2 5PT, UK.

Nicos Fotiadis, MD, PhD, Department of Interventional Radiology, Royal Marsden Hospital, 203 Fulham Rd, London SW3 6JJ, United Kingdom.

Corresponding author:

Ricardo Donners, MD

Department of Radiology, University Hospital Basel

Petersgraben 4, 4031 Basel, Switzerland

Telephone: +41 613286911

E-mail: [ricardo.donners@usb.ch](mailto:ricardo.donners@usb.ch)

Declaration of Interest:

The authors have no disclosures relevant to this manuscript.

**Abstract**

**Multiparametric bone MRI targeting aides lesion selection for CT-guided sclerotic bone biopsies in metastatic castrate resistant prostate cancer**

Background: Bone biopsies in metastatic castrate-resistant prostate cancer (mCRPC) patients can be challenging. This study’s objective was to prospectively validate a multiparametric bone MRI (mpBMRI) algorithm to facilitate target lesion selection in mCRPC patients with sclerotic bone disease for subsequent CT-guided bone biopsies.

*Methods:* 20 CT-guided bone biopsies were prospectively performed between 02/2021–11/2021 in 17 mCRPC patients with only sclerotic bone disease. Biopsy targets were selected based on MRI, including diffusion-weighted (DWI) and T1-weighted VIBE Dixon MR images, allowing for calculation of apparent diffusion coefficient (ADC) and relative fat-fraction (rFF), respectively. Bone marrow with high DWI signal, ADC <1100 µm^2^/s and rFF <20% was the preferred biopsy target. Tumor content and NGS-feasibility was assessed by a pathologist. Prognostic routine laboratory blood parameters, target lesion size, biopsy tract length, visual CT density, means of HU, ADC and rFF were compared between successful and unsuccessful biopsies (p<0.05 = significant).

*Results:* Overall, 17/20 (85%) biopsies were tumor-positive and next-generation genomic sequencing (NGS) was feasible in 13/18 (72%) evaluated samples. Neither laboratory parameters, diameter, tract length nor visual CT density grading showed significant differences between a positive versus negative or NGS feasible versus non-feasible biopsy results (each p>0.137). Lesion mean HU was 387±187 HU in NGS feasible and 493±218 HU in non-feasible biopsies (p=0.521). For targets fulfilling all MRI selection algorithm criteria, 13/14 (93%) biopsies were tumor-positive and 10/12 (83%) provided NGS adequate tissue.

Conclusions: Multiparametric bone MRI can facilitate target lesion selection for subsequent CT-guided bone biopsy in mCPRC patients with sclerotic metastases.

Trial registration: Committee for Clinical Research of the Royal Marsden Hospital registration number SE1220

Keywords:

Neoplasms; Image-Guided Biopsy; Computer Tomography, Genomics; Bone marrow

Abbreviations:

FO – fat-only

NGS – next-generation genomic sequencing

mCRPC – metastatic castrate-resistant prostate cancer

MpBMRI – Multiparametric bone MRI

rFF – relative fat-fraction

VIBE – volume interpolated breath-hold examination Dixon

WB – Whole-body

WO – water-only

**Introduction**

A significant proportion of metastatic castrate-resistant prostate cancer (mCRPC) patients harbor clinically actionable genome aberrations, which allow for targeted therapies (1). Tumor biopsies are essential for treatment decision-making based on individual tumor genomics. However, obtaining high quality tissue samples, containing sufficient tumor cells for advanced molecular analyses, such as next-generation genomic sequencing (NGS), can be challenging in mCRPC patients.

In mCRPC, bone is by far the most common metastatic site and up to 50% of patients show only skeletal disease (2-4) . This is problematic, as bone metastases are often viewed as suboptimal biopsy targets compared with soft-tissues (5). Even when performed under CT-guidance, there is a significant chance that a bone biopsy will be tumor-negative or contain only few tumor cells, omitting NGS (5-9).

There are no established guidelines for optimal biopsy target selection in mCRPC patients with bone metastases. Generally, lytic (low CT-attenuation) lesions are preferred over sclerotic (high CT-attenuation) lesions (6, 7, 9-11). However, the former are less common in mCRPC (4). Reported biopsy success rates for malignant tumor diagnosis and genomic sequencing from sclerotic prostate cancer bone metastases are relatively poor, ranging between 33% and 54% (5, 8, 9). This warrants further research for improvement of these results.

Imaging plays a key role for biopsy target lesion selection, but CT lacks specificity for discrimination between active and treated sclerotic bone disease. MRI on the other hand, is the gold standard for assessment of bone marrow conditions. In a retrospective study, (12)a simple multiparametric bone MRI (mpBMRI) algorithm using diffusion-weighted MRI (DWI) signal intensity (SI), apparent diffusion coefficient (ADC) and relative fat-fraction (rFF) percentages reliably identified active bone metastases yielding a tumor-positive and NGS feasible biopsy result (12).

The purpose of this study was to test and validate the mpBMRI target lesion selection algorithm in a prospective setting to facilitate target lesion selection for subsequent CT-guided bone biopsies in mCRPC patients with sclerotic disease.

**Material and Methods**

This prospective study was approved by the institutional review board and performed as part of multiple phase I and II clinical trials, which required tumor biopsies and were run at our institution. Biopsies were obtained as per respective trial protocol and no additional interventions were performed nor extra samples obtained for this bone biopsy study. Each patient provided written informed consent for trial inclusion, the CT-guided bone biopsy and research related use of medical data.

*Patients*

Patients were consecutively recruited between 15/02/2021 and 10/11/2021. Study inclusion criteria were: mCRPC with a histopathological diagnosis, patients were fit for CT-guided bone biopsy procedure and MRI, presence of accessible, sclerotic bone lesions, and availability of written informed consent. Exclusion criteria were: lack of fitness for CT-guided bone biopsy, contraindications to percutaneous biopsy according to contemporary guidelines with particular attention to coagulation status (13, 14), general MRI contraindications and lack of sclerotic bone lesions. When patients had accessible soft-tissue or lytic bone metastases, these were chosen as biopsy targets regardless of the presence of sclerotic bone disease and patients were excluded from this study.

Patient blood parameters obtained within four weeks prior to biopsy, including alkaline phosphatase (ALP) in Units/L, lactate dehydrogenase (LDH) in Units/L, hemoglobin (Hb) in g/L, platelets x10^9^/L, International Normalized ratio (INR) and prostate specific antigen (PSA) levels in µg/L as well as Gleason scores were recorded.

*Imaging*

Whole-body (WB) mpBMRI, including free-breathing DWI and breath-hold T1-weighted 2-point volume interpolated breath-hold examination (VIBE) Dixon sequences, was acquired as part of the regular disease staging protocol for each patient. When WB-mpBMRI within 3 months prior to the planned biopsy procedure was not available, dedicated limited field-of-view mpBMRI of the likely biopsy target site (such as the pelvis) determined by available imaging, including prior WB-mpBMRI studies, was performed. Sequence parameters for the institute’s 1.5T MRI scanner (MAGNETOM Sola, Siemens Healthineers) are summarized in **Table 1**. From the VIBE Dixon fat-only (FO) and water-only (WO) images, relative fat fraction (rFF) images were calculated as rFF = FO/(FO+WO) (**Figure 1**).

*Imaging evaluation and target lesion selection*

A diagnostic and interventional radiology fellow with three years of experience in mpBMRI of malignant bone disease, competent in performing CT-guided bone biopsies, evaluated all imaging prior to the biopsy. MpBMRI was scrutinized for bone metastases in accessible location, which would be recommended as biopsy targets according to the bone biopsy lesion selection algorithm. Bone biopsies in lesions with high DWI signal, mean ADC < 1000 µm^2^/s and mean rFF < 20% were selected to offer high diagnostic yield and sufficient material for NGS (12). Consequently, a target lesion deemed likely to provide high tumor-tissue yield needed to fulfill all of the following MRI criteria:

1. lack of suppression i.e. high signal on DWI
2. mean ADC < 1100 µm^2^/s
3. mean rFF < 20%

The target selection process for a patient with multiple sclerotic bone lesions is visualized in **Figure 2.** When no accessible metastasis fulfilling all three mpBMRI selection criteria was available, a high DWI signal bone lesion with ADC and rFF values close to the desired thresholds was selected for biopsy. Targets were chosen in consensus with a senior consultant radiologist, with 10-years of experience in mpBMRI of mCRPC. Target lesions were highlighted on the department’s picture archiving and communication systems (PACS, IDS7 Version 22.1, Sectra, Sweden). To minimize procedural risks, pelvic ring lesions were preferred when possible.

*Biopsy procedure*

A specialized interventional radiologist, who had been performing CT-guided bone biopsies for 15 years or a supervised interventional radiology fellow, performed the biopsies included in this study. MpBMRI with the previously determined target lesion suggestion was reviewed again immediately before the procedure.

Patients provided written informed consent for the CT-guided bone biopsy and possible complications and concerns were discussed. The patient was placed in the CT gantry (Somatom Definition Edge, Siemens Healthineers). Positioning was adapted according to the target site, optimizing patient comfort where possible.

The WHO checklist was completed and the procedure site was marked on the patient skin, placing an x-ray grid for orientation. A 3 mm thin slice CT scout encompassing the grid was performed and the needle access position was planned. Patients received 100% oxygen via nasal cannula and intravenous conscious sedation using midazolam and fentanyl under constant monitoring of vital parameters.

Lidocaine 1% was applied to the skin and periosteum with CT imaging confirming the correct needle path as planned to the biopsy target. Madison™ (Merit Medical, USA) 11G and 13G biopsy needle systems were used. Bone biopsies were performed under CT-guidance. Biopsy samples were handed to the pathologist and fixed. The biopsy needle systems were removed, dressing was applied to the access site, and a spiral CT scan was performed to rule out immediate complications (**Figure 3)**. Patients remained under surveillance with bed rest for three hours in the recovery area following the procedure and were then discharged safely home.

Needle gauge (G), length of the biopsy tract and number of bone biopsy samples obtained were recorded. The former was determined by the interventionalist prior to the procedure, with a general preference for a larger diameter needle to provide larger samples. For smaller bone calibre and areas with greater potential risk, such as the sternum, a smaller calibre needle was chosen. The number of biopsy samples was determined by the present member of the pathology team, instructing on the specific number of samples required per patient as per trial protocol as well as by the interventionalist, who performed a visual inspection of the samples. After the biopsy cores were obtained, samples were fixed for 24-30 hours and decalcified in an EDTA solution for two days. Bone biopsy samples were deemed suitable for NGS when showing at least 150 cancer cells on H&E stained, 2 µm thick specimen slices. 10 x 6 µm sections were prepared for sequencing. Biopsy specimens containing less than 150 tumor cells on H&E stained slices were deemed not suitable for NGS by the pathologist. When NGS was performed, presence of mutations and copy number variations were recorded.

*Statistics*

Statistical analyses were performed using commercially available software (IBM SPSS Statistics Version 25, IBM Corp. Armonk, New York, USA). Qualitative, nominal scaled variables were compared using chi-squared tests. Quantitative, continuous variables were compared using Mann-Whitney-U tests. A p-value < 0.05 was deemed statistically significant.

**Results**

Seventeen mCRPC patients with exclusively sclerotic bone metastases underwent 20 CT-guided bone biopsies and were prospectively included in this study. No procedure-related complications were encountered. Median age of the study population was 69 years (range 53 – 83 years) at the time of biopsy.

Median time interval between mpBMRI and CT-guided bone biopsy was 26 days. 17/20 (85%) biopsies were tumor-positive. Two samples were not evaluated for NGS feasibility as NGS results were already available from earlier biopsies. In total, NGS was feasible in 13/18 (72%) tissue samples. Five biopsies provided insufficient tumor-cell content NGS results were available for 12 biopsies, as the remaining sample was deemed feasible, but not analyzed.

NGS identified tumor genome mutations in 10/12 (83%) samples, functional importance was rated as pathogenic in 8 samples according to the ClinVar or American College of Medical Genetics and Genomics reporting recommendations. Copy number genome variations were identified in 7 (58%) cases **(Table 2)**.

11G bone biopsy needles were used in 18/20 biopsies and 13G needles for the remaining two. Between two and six samples were obtained for each biopsy, the median was three biopsy cores.

*Laboratory parameters*

Median values of laboratory parameters are summarized in **Table 3**. There was no significant difference between laboratory parameter measurements in tumor-positive compared with negative biopsies (each p > 0.266) nor between NGS-feasible and non-feasible biopsies (each p > 0.153).

*Lesion diameter and biopsy tract length*

There were no significant differences in lesion diameter between tumor-positive (mean 22 mm) and negative biopsies (mean 47 mm, p = 0.152) nor between NGS-feasible (mean 23 mm) and non-feasible biopsies (39 mm, p = 0.401). Analogously, no significant differences were found regarding biopsy tract length in tumor-positive (mean 17 mm) and negative (mean 25 mm, p = 0.222) nor between NGS-feasible (mean 17 mm) and non-feasible (mean 25 mm, p = 0.322) biopsies.

*CT lesion appearance and attenuation*

Twelve biopsied lesions were classified as predominantly mildly sclerotic and eight as predominantly densely sclerotic. Overall, 11/12 (92%) mildly sclerotic biopsies and 6/8 (75%) biopsies of predominantly densely sclerotic lesions were tumor-positive (p = 0.306). NGS was feasible in 10/12 (83%) mildly sclerotic lesions and 3/5 (60%) densely sclerotic lesions (p = 0.137).

Mean HU of lesions with tumor-positive biopsies was 398 ± 190 HU and 483 ± 181 HU in tumor-negative biopsies (p = 0.711). Regarding NGS feasibility, mean HU was 387 ± 187 HU in NGS-feasible and 493 ± 218 HU in lesions with NGS non-feasible biopsies (p = 0.521).

*Multiparametric bone MRI target lesion selection*

Lesions were only chosen for biopsy, when failing to suppress and consequently showing high signal on DWI. Quantitative mpBMRI mean ADC and mean rFF percentage measurements for all included biopsies are shown in **Table 4**.

Overall, 14/20 biopsies fulfilled all of the three criteria of the target lesion selection algorithm (high DWI SI, ADC < 1100 µm^2^/s, rFF < 20%). Among these lesions, 13/14 (93%) biopsies were tumor-positive and 10/12 (83%) provided sufficient material for NGS. This includes eleven mildly and three densely sclerotic lesions. Six bone marrow lesions lacked the “adequate target” mpBMRI selection criteria, and displayed mean rFF slightly larger than 20% (range 21% – 25%). Among these, 4/6 (60%) biopsies were tumor-positive and 3/6 (50%) biopsies provided sufficient malignant tumor cells for NGS.

**Discussion**

In this prospective proof-of-principle pilot study we have shown that utilizing mpBMRI for sclerotic bone target selection in mCRPC patients for subsequent CT-guided biopsyresults in high biopsy success rates. 85% of biopsies were tumor-positive and NGS feasibility was 72%. Among lesions which fulfilled all three mpBMRI target selection criteria, namely high DWI signal, ADC < 1100 µm/s^2^ and rFF < 20%, 93% of biopsies were tumor-positive and the NGS feasibility rate was 83%.

In previous studies, a 53% tumour-positive and 45% sequencing success rate were described for CT-guided sclerotic bone biopsies (8, 9). By comparison, the tumour-positivity (85%) and NGS feasibility rate (72%) were substantially improved in our prospective pilot cohort.

Holmes et al. described significantly improved bone biopsy success in predominantly radiolucent compared with sclerotic lesions, with 18/19 (95%) lytic lesion biopsies being tumor-positive (8). By comparison, only 20/38 (53%) sclerotic bone biopsies were tumor-positive, including 5/15 (33%) predominantly densely sclerotic and 15/23 (65%) predominantly subtle sclerotic lesions. Other researchers found 50% of all bone biopsy cores in 23 prostate cancer patients to be tumour positive and RNA isolation feasible in 14/31 (45%) of prostate cancer sclerotic bone biopsies(5, 9). Radiolucent, lytic bone metastases are generally chosen over sclerotic bone metastases for CT-guided biopsies (6, 7, 16). However, lytic bone metastases are often absent in mCRPC patients (4). For this reason and to provide clinical evidence for mCRPC patients with exclusively sclerotic disease, we excluded lytic metastases from the presented study. Using a previously established mpBMRI targeting algorithm, utilizing DWI SI, ADC and rFF, we achieved very good diagnostic and NGS success rates, while only including sclerotic bone biopsies. Our approach led to some improvement over the previously published CT-guided bone biopsy diagnostic (85% versus 76%) and NGS (72% versus 71%) success rates in cancer patients at our institution (12). Several factors may account for lack of a more substantial increase in biopsy success when comparing this prospective study with the previously published retrospective analysis in our department: First and foremost, the previous analysis was performed independently of CT appearance. It was not limited to sclerotic bone lesions, but included lytic disease as well. Additionally, the 76% tumor-positive and 71% NGS feasibility rate in this previous study were already towards the upper end of published biopsy success rates and limit room for improvement.

More recently, studies suggested that PSMA targeting may improve tumor-tissue yield from CT-guided bone biopsies in prostate cancer patients, but CT attenuation of biopsied lesions was not reported in the available studies and needs to be evaluated in the future (17, 18).

Overall, our study results validate the use of mpBMRI for target lesion selection in mCRPC patients with sclerotic bone metastases. Further research to permit translation of our findings to other centers, especially with regard to the reproducibility of quantitative mpBMRI target selection thresholds is needed. Eventually, our suggested approach could lead to a paradigm shift for mCRPC patients with only sclerotic bone metastases: Sclerotic bone disease is commonly regarded as an unsuitable biopsy target for obtaining tumor samples adequate for NGS. The increased tumor tissue yield when using mpBMRI for biopsy target selection may allow these patients to benefit from personalised oncology treatments, including biomarker-driven and molecularly targeted therapies. NGS revealed somatic genome mutations or copy number alterations in all analyzed samples, highlighting the potential patient benefit.

We recognize the small study population as a major limitation for this prospective study. However, previously published prospective CT-guided bone biopsy studies aiming to optimize target lesion selection feature similar size study cohorts. Published analyses of sclerotic bone lesion biopsies in less than 30 patients are frequently used for reference and decision-support (7, 9). The comparison with these previous studiessuggests superiority of chosing targets using mpBMRI. As mpBMRI is now part of our preinterventional work-up, no comparison cohort was available. We suggest using mpBMRI to facilitate target lesions selection for subsequent CT-guided biopsies. There may be a role for MRI-guided procedures, allowing for improved visualisation of marrow lesions. Lesion selection parameters, especially the ADC, depend on the imaging parameters including the selection of b-values. While higher b-values may improve lesion conspicuity they lead to decreased SNR and image quality. The protocol presented in this study adheres to the contemporary recommendation for WB-MRI of prostate cancer bone disease (19). We believe our results supply principal evidence in favor of using mpBMRI to optimize CT-guided bone biopsy target lesion selection applicable to a wider population of prostate cancer patients. This was a single center study. Larger multi-center studies, optimizing parameters across sites and hardware vendors are needed to achieve sufficient technical validation.

**Conclusion**

In conclusion, mpBMRI can facilitate target selection in mCPRC patients with sclerotic bone metastases for subsequent CT-guided biopsy. This approach resulted in high diagnostic biopsy yield and feasibility of NGS in this patient group. We recommend mpBMRI as part of the pre-interventional imaging work-up.

**Declarations:**

Ethical approval and consent to participate

This bone biopsy study was approved by the local research and ethics committee, Committee for Clinical Research of the Royal Marsden Hospital registration number SE1220. Informed consent was obtained from each participant.

Consent for publication

Consent for publication was obtained when obtaining written informed consent from each included individual.

Availability of data and materials

Study data are available upon reasonable request.

Competing interests

The authors are not aware of any competing interests relevant to this manuscript.

Funding information:

Research funding for this work was received from Prostate Cancer UK, the Movember Foundation through the London Movember Centre of Excellence (CEO13_2-002), the Prostrate Cancer Foundation, Cancer Research UK (Centre Programme grant) and Experimental Cancer Medicine Centre grant funding from Cancer Research UK. This research was also partly supported by the “Foundation of the Swiss Society of Radiology for Research, Postgraduate and Continuing Medical Education” and “Research Fund for excellent Junior Researchers of the University of Basel”.

Author’s contributions

RD – Conceptualization; Data curation; Formal analysis; Funding acquisition; Investigation; Methodology; Project administration; Resources; Software; Supervision; Validation; Visualization; Writing – original draft; Writing – review & editing.

IF - Data curation; Formal analysis; Investigation; Software; Validation; Visualization; Writing – review & editing.

DW - Project administration; Validation; Writing – review & editing.

NT - Conceptualization; Data curation; Formal analysis; Funding acquisition; Methodology; Software; Supervision; Visualization; Writing – review & editing.

DMK - Conceptualization; Funding acquisition; Project administration; Resources; Writing – review & editing.

SC - Data curation; Formal analysis; Investigation; Project administration; Validation; Writing – review & editing.

JSB - Project administration; Resources; Writing – review & editing.

NF - Conceptualization; Data curation; Formal analysis; Funding acquisition; Investigation; Methodology; Project administration; Resources; Software; Supervision; Validation; Writing – review & editing.

Acknowledgements

Not applicable.

**References**

1. Bubendorf L, Schöpfer A, Wagner U, Sauter G, Moch H, Willi N, et al. Metastatic patterns of prostate cancer: an autopsy study of 1,589 patients. Hum Pathol. 2000;31(5):578-83.

2. Coleman RE. Metastatic bone disease: clinical features, pathophysiology and treatment strategies. Cancer Treat Rev. 2001;27(3):165-76.

3. Coleman RE, Rubens RD. The clinical course of bone metastases from breast cancer. Br J Cancer. 1987;55(1):61-6.

4. Coleman RE, Croucher PI, Padhani AR, Clézardin P, Chow E, Fallon M, et al. Bone metastases. Nat Rev Dis Primers. 2020;6(1):83.

5. McKay RR, Zukotynski KA, Werner L, Voznesensky O, Wu JS, Smith SE, et al. Imaging, procedural and clinical variables associated with tumor yield on bone biopsy in metastatic castration-resistant prostate cancer. Prostate Cancer Prostatic Dis. 2014;17(4):325-31.

6. Li Y, Du Y, Luo TY, Yang HF, Yu JH, Xu XX, et al. Factors influencing diagnostic yield of CT-guided percutaneous core needle biopsy for bone lesions. Clin Radiol. 2014;69(1):e43-7.

7. Wu JS, Goldsmith JD, Horwich PJ, Shetty SK, Hochman MG. Bone and soft-tissue lesions: what factors affect diagnostic yield of image-guided core-needle biopsy? Radiology. 2008;248(3):962-70.

8. Holmes MG, Foss E, Joseph G, Foye A, Beckett B, Motamedi D, et al. CT-Guided Bone Biopsies in Metastatic Castration-Resistant Prostate Cancer: Factors Predictive of Maximum Tumor Yield. J Vasc Interv Radiol. 2017;28(8):1073-81.e1.

9. Spritzer CE, Afonso PD, Vinson EN, Turnbull JD, Morris KK, Foye A, et al. Bone marrow biopsy: RNA isolation with expression profiling in men with metastatic castration-resistant prostate cancer--factors affecting diagnostic success. Radiology. 2013;269(3):816-23.

10. Hao DJ, Sun HH, He BR, Liu TJ, Jiang YH, Zhao QP. Accuracy of CT-guided biopsies in 158 patients with thoracic spinal lesions. Acta Radiol. 2011;52(9):1015-9.

11. Hwang S, Lefkowitz RA, Landa J, Zheng J, Moskowitz CS, Maybody M, et al. Percutaneous CT-guided bone biopsy: diagnosis of malignancy in lesions with initially indeterminate biopsy results and CT features associated with diagnostic or indeterminate results. AJR Am J Roentgenol. 2011;197(6):1417-25.

12. Donners R, Figueiredo I, Tunariu N, Blackledge M, Koh DM, de la Maza MLDF, et al. Multiparametric bone MRI can improve CT-guided bone biopsy target selection in cancer patients and increase diagnostic yield and feasibility of next-generation tumour sequencing. Eur Radiol. 2022.

13. Patel IJ, Davidson JC, Nikolic B, Salazar GM, Schwartzberg MS, Walker TG, et al. Consensus guidelines for periprocedural management of coagulation status and hemostasis risk in percutaneous image-guided interventions. J Vasc Interv Radiol. 2012;23(6):727-36.

14. Veltri A, Bargellini I, Giorgi L, Almeida PAMS, Akhan O. CIRSE Guidelines on Percutaneous Needle Biopsy (PNB). Cardiovasc Intervent Radiol. 2017;40(10):1501-13.

15. Moher D, Liberati A, Tetzlaff J, Altman DG, Group P. Preferred reporting items for systematic reviews and meta-analyses: the PRISMA statement. BMJ. 2009;339:b2535.

16. Sailer V, Schiffman MH, Kossai M, Cyrta J, Beg S, Sullivan B, et al. Bone biopsy protocol for advanced prostate cancer in the era of precision medicine. Cancer. 2018;124(5):1008-15.

17. de Jong AC, Smits M, van Riet J, Fütterer JJ, Brabander T, Hamberg P, et al. Ga-PSMA-Guided Bone Biopsies for Molecular Diagnostics in Patients with Metastatic Prostate Cancer. J Nucl Med. 2020;61(11):1607-14.

18. van Steenbergen TRF, Smits M, Scheenen TWJ, van Oort IM, Nagarajah J, Rovers MM, et al. Ga-PSMA-PET/CT and Diffusion MRI Targeting for Cone-Beam CT-Guided Bone Biopsies of Castration-Resistant Prostate Cancer Patients. Cardiovasc Intervent Radiol. 2020;43(1):147-54.

19. Padhani AR, Lecouvet FE, Tunariu N, Koh DM, De Keyzer F, Collins DJ, et al. METastasis Reporting and Data System for Prostate Cancer: Practical Guidelines for Acquisition, Interpretation, and Reporting of Whole-body Magnetic Resonance Imaging-based Evaluations of Multiorgan Involvement in Advanced Prostate Cancer. Eur Urol. 2017;71(1):81-92.

**Tables**

Table 1: Diffusion-weighted and Dixon MRI parameters

| **Parameter** | **DWI** | **T1-weighted VIBE Dixon** |
| --- | --- | --- |
| Plane | axial | axial |
| Slice thickness (mm) | 5 | 5 |
| b-values | 50, 600, 900 | - |
| Field of view (mm) | 430 x 390 | 430 x 390 |
| Acquisition matrix | 134 x 108 | 256 x 180 |
| Repetition time (ms) | 6240 | 7.14 |
| Echo time (ms) | 73 | 2.39 |
| Number of averages | 3 – 3 – 6 (b50, b600, b900) | 1 |
| Flip angle | 90° | 20° |
| Bandwidth (Hz/pixel) | 1964 | 470 |
| Acquisition time (min:s) | 3:51 | 0:19 |

VIBE – volume-interpolated breath-hold examination

Table 2: Biopsy next-generation genomic sequencing results

| StudyID | **Somatic Mutations** | | | **Copy Number Variations** |
| --- | --- | --- | --- | --- |
|  | *Gene* | *Mutation* | *Functional Impact* |  |
| 2 | SRC  TP53 | p.P171L  p.R158H | Uncertain  Uncertain | AR amplification |
| 5 | EGFR  NOTCH2  TP53 | p.A289V  p.C342R  p.M237I | Uncertain  Likely pathogenic  Pathogenic | MAP2K1, PTEN deep deletion AR, FANCB amplification |
| 6 | CDK12 | p.G162W | Uncertain | MAP2K1, PTEN deep deletion  AR, FANCB amplification |
| 9 | TP53 | c.258+1G>A | Pathogenic | - |
| 10 | FANCG  WT1 | C308-1G  p.H359D | Pathogenic  Uncertain | - |
| 12 | - |  |  | PIK3R1, PTEN deep deletion |
| 14 | - |  |  | MAP2K4, NOTCH1, PTEN deep deletion  AR amplification |
| 15 | PIK3CA  FANCA | p.E545K  p.S132R | Pathogenic  Uncertain | - |
| 16 | PDGFRA  TP53 | p.I317T  p.R273C | Uncertain  Pathogenic | - |
| 17 | XPC | c.104-2A>G | Pathogenic | - |
| 19 | AR  ATM  BLM  FANCC  FANCF  MAP3K1  MSH6  RECQL4 | p.T878A  p.T1743I  p.F663I  p.R555Q  p.A186V  p.D1170del  p.R1334W  p.G120R | Pathogenic  Likely pathogenic  Uncertain  Likely benign  Benign  Uncertain  Pathogenic  Likely benign | AR, ATRX, RECQL4 amplification |
| 20 | BRCA2  PIK3CA  TP53 | p.I1859Kfs*3  p.E545K  p.R282W | Pathogenic  Pathogenic  Pathogenic | AR amplification |

Functional impact of each somatic mutation is rated as per ClinVar and American College of Medical Genetics and Genomics reporting recommendations, AR – androgen receptor, PTEN – phosphatase and tensin homologue

Table 3: Median routine blood parameters and tumor markers

|  | **Tumor** | | | **NGS** | | |
| --- | --- | --- | --- | --- | --- | --- |
| **Parameter** | *Positive* | *Negative* | *P* | *Feasible* | *Not-feasible* | *P* |
| **AlkPh U/L** | 195 | 274 | 0.266 | 267 | 191 | 0.588 |
| **LDH U/L** | 241 | 182 | 0.634 | 241 | 182 | 0.153 |
| **HB g/L** | 110 | 119 | 0.874 | 119 | 110 | 0.323 |
| **Platelets x 10^9^/L** | 268 | 255 | 0.791 | 268 | 321 | 0.301 |
| **PSA µg/L** | 244 | 198 | 0.705 | 244 | 129 | 0.391 |
| **Gleason** | 7.5 | 7 | 0.925 | 8 | 7.5 | 1.000 |

NGS – Next-generation genomic sequencing, AlkPH - alkaline phosphatase, LDH - lactate dehydrogenase, HB – hemoglobin, PSA – prostate specific antigene, P < 0.05 = significant

Table 4: Multiparametric bone MRI ADC and relative fat fraction (rFF) percentage values of the study cohort

| **Study ID** | **Site** | **Mean ADC in µm^2^/s** | **Mean rFF in %** | **mpbRMI target recommendation** | **Tumor** | **NGS** |
| --- | --- | --- | --- | --- | --- | --- |
| 1 | Os ilium | 567 | 25 | No | negative | not feasible |
| 2 | Os ilium | 855 | 18 | Yes | positive | feasible |
| 3 | Os pubis | 846 | 24 | No | positive | not feasible |
| 4 | Sternum | 664 | 13 | Yes | positive | n.a |
| 5 | Os ilium | 706 | 8 | Yes | positive | feasible |
| 6 | Os ilium | 736 | 11 | Yes | positive | feasible |
| 7 | Sternum | 720 | 12 | Yes | positive | n.a. |
| 8 | Lumbar vertebra | 610 | 21 | No | negative | not feasible |
| 9 | Sternum | 724 | 18 | Yes | positive | feasible |
| 10 | Os ilium | 567 | 23 | No | positive | feasible |
| 11 | Os ilium | 701 | 11 | Yes | negative | not feasible |
| 12 | Lumbar vertebra | 679 | 22 | No | positive | feasible |
| 13 | Os ilium | 877 | 11 | Yes | positive | feasible |
| 14 | Os ilium | 882 | 8 | Yes | positive | feasible |
| 15 | Os ilium | 567 | 23 | No | positive | feasible |
| 16 | Os ilium | 762 | 11 | Yes | positive | feasible |
| 17 | Os ilium | 764 | 11 | Yes | positive | feasible |
| 18 | Lumbar vertebra | 542 | 19 | Yes | positive | not feasible |
| 19 | Os ilium | 955 | 18 | Yes | positive | feasible |
| 20 | Os ilium | 910 | 19 | Yes | positive | feasible |

rFF – relative fat fraction, mpBMRI – multiparametric bone MRI, NGS – next-generation genomic sequencing


**Figures**

**Figure 1:** Multiparametric bone MRI imaging protocol for CT-guided bone biopsy target lesion selection including DWI and T1-weighted, gradient-echo Dixon images, CT of the same area for comparison, FO – fat-only Dixon image, WO – water-only Dixon image, rFF – relative fat fraction, b900 – high b-value DWI with corresponding ADC map

**Figure 2:** CT-guided bone biopsy target lesion selection process based on multiparametric bone MRI, considering high b-value DWI (b900) signal intensity (SI) ADC and rFF in a metastatic castrate resistant 74-year-old prostate cancer patient with multifocal sclerotic bone metastases, CT images for comparison

The anterior metastasis in the first row (arrow) shows low DWI SI, the posterior lesion (arrowhead) displays high ADC (>1100 µm^2^/s) and high rFF (>20%) and was thus not fit for biopsy

The pubic bone metastasis in the second row shows high DWI SI, ADC < 1100 µm^2^/s and rFF <20% and was biopsied, biopsy provided sufficient malignant tumor cells for next-generation genomic sequencing

The ischial metastases in the third row displays high DWI signal, an ADC < 1100 µm^2^/s, but an rFF of 31% and was thus not considered for biopsy

**Figure 3:** CT-guided bone biopsy in a 86-year old prostate cancer patient, CT before biopsy (CTpre) displays diffuse sclerosis, the chosen target area according to the b900 DWI, ADC and rFF is indicated by the arrow, the biopsy-CT (CT-Bx) and post-interventional control spiral (CTpost) show biopsy needle and biopsy tract in the target area, note how the majority of the sclerotic pelvic bone displays high signal on the rFF image

Supplementary material

Supplemetary table 1: CT-guided, sclerotic bone biopsies in prostate cancer patients, literature results

| **Authors** | **Year** | **N** | **Available imaging** | **Lesion selection** | **Biopsy result** |
| --- | --- | --- | --- | --- | --- |
| Spritzer et. al.(9) | 2013 | 31 | CT, bone scan | Low attenuation lesions preferred, periphery of high attenuation lesions targeted | 14/31 (45%) RNA isolation |
| McKay et. al.(5) | 2014 | 23 | CT, bone scan | Clinical judgement* | 50% cores tumour-positive |
| Holmes et. al. (8) | 2017 | 38 | CT, bone scan | Clinical judgement* | 20/38 (53%) tumour-positive |

N – number of CT-guided sclerotic bone lesion biospies, * biopsy target lesions were chosen based on clinical judgement of the interventional radiologist, not further specified
